# A case of fulminant sepsis caused by *Capnocytophaga canimorsus* after a dog bite

**DOI:** 10.1016/j.idcr.2020.e00798

**Published:** 2020-05-11

**Authors:** Piotr Woźniak, Robert Szymczak, Agata Piotrowska

**Affiliations:** Department of Emergency Medicine, Medical University of Gdansk, Poland

**Keywords:** Sepsis, Septic shock, Dog bite, Capnocytophaga, Canimorsus

## Abstract

Many species of microorganisms of various human pathogenicity have been identified in the oral cavities of dogs and cats. One of them is *Capnocytophaga canimorsus*, a Gram-negative bacterium of the *Flavobacteriacae* family, with unique abilities to forage sugars from host tissues and avoid the host immune response. Although *C. canimorsus* may be isolated from the oral cavities of most dogs and cats, severe human infection is very rare according to studies (0.67 cases/million/year). A canine or feline bite is the most common source of infection. At the highest risk are asplenic or functionally asplenic patients as well as individuals with cirrhosis or a history of alcohol abuse. We report a fatal case of *C. canimorsus* sepsis in a patient with a spleen.

## Introduction

In the gastrointestinal tract of a common dog or cat there are numerous species of microorganisms of various pathogenicity for humans. One of them is *Capnocytophaga canimorsus*, a Gram-negative, facultatively anaerobic bacterium of the *Flavobacteriacae* family. We present the case of a female patient who presented with this zoonotic infection. The literature states that even though *C. canimorsus* may be isolated from the oral cavity of 67–86% of dogs and 55–84% of cats [[1], [2], [3], [4]], severe human infection is extremely rarely reported (0.67 cases/million/year according to the only available register maintained in the Netherlands) [5]. Infection in humans is usually a consequence of a bite or scratch by a dog or, less commonly, a cat. At the highest risk are asplenic or functionally asplenic patients as well as individuals with cirrhosis or a history of alcohol abuse [[5], [6], [7]]. The course of infection can be dramatic and takes the form of fulminant septicemia with hypoglycemia, shock, purpura, DIC and multiple organ failure [6,8,9]. Microbiological diagnosis may be difficult due to a slow culture growth of up to 14 days, if the bacteria are not recognized on a peripheral smear [8]. Therapy consists of beta-lactam antimicrobials. The fatality rate associated with severe *Capnocytophaga Canimorsus* infection amounts to 28–30% and is high even among patients without compromised immunity [6,8,10].

## Case study

A 59-year-old female patient was brought to the Emergency Department of the University Clinical Centre in Gdansk by ambulance, from the Admission Room of a neighboring hospital of infectious diseases, due to a dynamically deteriorating condition, involving signs of septic shock. The patient also was found to have severe hypoglycemia (31 mg/dL) despite no known antihyperglycemic therapy. Before the patient was transferred to the ED, she had a blood culture taken and an initial dose of a wide spectrum antimicrobial (ceftriaxone 2 g IV). She was also given 2000 mL of crystalloids IV, 40% glucose infusion solution, 200 mg of hydrocortisone IV, CaCl_2_, MgSO_4_ and morphine. The history taken from the patient and her daughter in the Emergency Room included about 3 days of progressive weakness, abdominal pain and diarrhea. A history was also obtained of a superficial dog bite on the left hand that had happened 3 days previously. Her history of chronic conditions included only arterial hypertension and alcohol abuse at some point in the past, but without clear medical alcohol misuse diagnosis. Due to the lack of an Intensive Care Unit in that hospital, the patient was referred to the Emergency Room of a neighboring University Hospital.

**Fig. 1:**
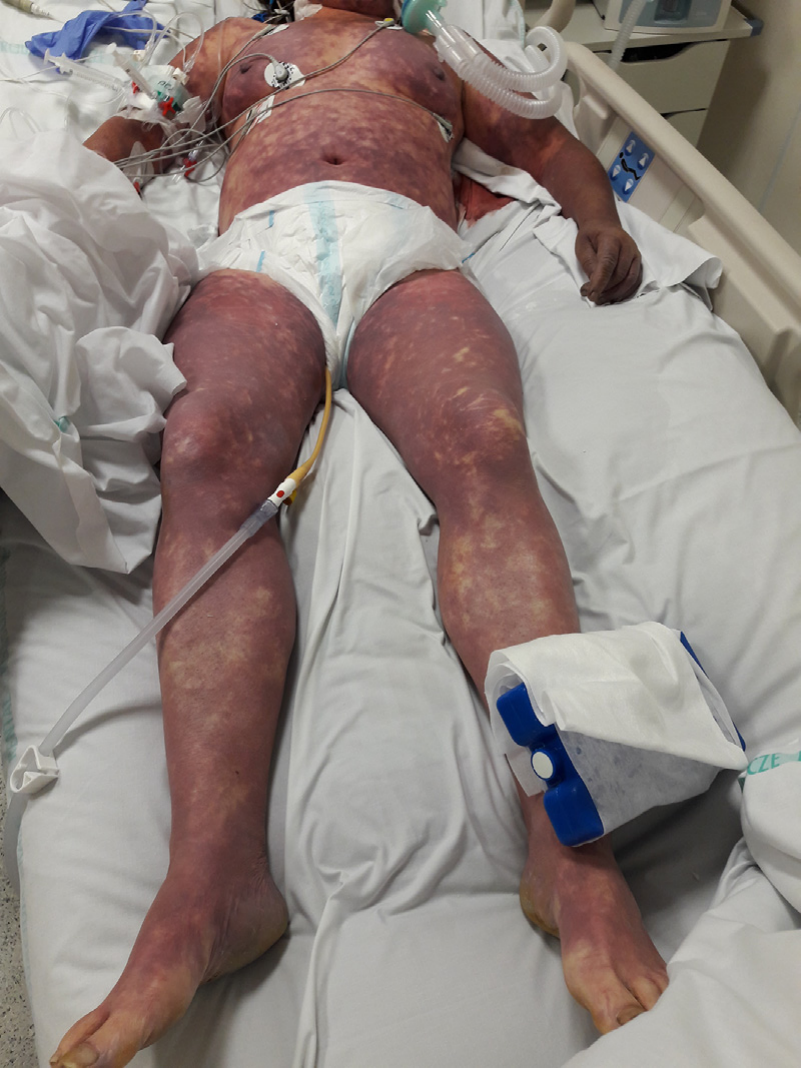
coagulopatic skin lesions.

On admission to our Emergency Room the patient was conscious, maintained logical verbal contact, although she presented with hyperventilation, visible peripheral cyanosis, signs of blood flow centralization, shock and coagulopathy. The patient presented with extensive extravasations and purpuric skin lesions covering most of the body. We also observed bleedings at the sites of peripheral intravenous cannulas insertion (Fig. 1). Vital signs showed noninvasive arterial blood pressure NIBP: 70/40 mmHg, tachycardia 120 bpm, fever 39 °C, peripheral saturation SpO2: 92% during passive oxygen therapy 5 L/min.

Point of Care biochemistry parameters measured on admission indicated metabolic acidosis. pH: 7.24; *p*CO_2_: 29 mmHg; *p*O_2_: 82 mmHg; BE: −13.8 mmol/L; serum lactates 6 mmol/L.

The patient was sedated, endotracheally intubated (following premedication with propofol) and mechanically ventilated. Due to suspected septic shock, we immediately collected another blood culture sample (no urine sample was available due to anuria). Fluid resuscitation was initiated: crystalloid solution infusion and a wide spectrum antibacterial administered according to the local protocol for septic shock: piperacillin + tazobactam 4.5 g IV.

Despite intensive fluid resuscitation, hemodynamic stability could not be regained, so we first initiated an intravenous infusion of dopamine followed by noradrenaline. Laboratory test results confirmed the initial diagnosis of septic shock with evidence of multiple organ failure and coagulopathy (Table 1). There were no parenchymal lesions found in the chest X-ray. Blood cultutres were reported to show Gram-negative rods. We administered hydrocortisone, diuretics and omeprazole, and transfused plasma (FFP) as well as platelet concentat (PC) units. Despite intensive care the patient’s condition deteriorated and 16 h after admission she had cardiac arrest with asystole. Due to exhaustion of therapeutic options, the patient was declared dead. The necropsy protocol (Table 2) confirmed shock, most probably septic, as the cause of death with no confirmed chronic liver disease as well as no chronic spleen abnormalities.

**Table 1:** laboratory test results.

CRP	PCT	WBC	Hb	PLT	GFR	Crea	D-Dimers	INR	APTT	CKMB	hsTnI
288.3 (<5.0)	74.9 (<0.5)	3.61 (4−10)	13.0 (12.0−15.0)	17 (150−410)	16(>60)	3.07 (0.55−1.02)	43,953 (<500)	2.69 (0.9−1.3)	3.51 (0.9−1.2)	9.4 (<6.6)	0.109

**Table 2:** Conclusion of the necropsy protocol.

Necropsy protocol:
Immediate cause of death: shock, most probably septic shock. In the course of shock, the following occurred: acute multi organ failure of kidneys (acute tubular necrosis), lungs (oedema and fluid in the pleural cavities), liver (centrilobular necrosis) and adrenal glands (coagulative necrosis). The patient’s history was positive for generalized atherosclerosis and left ventricle hypertrophy, most probably due to chronic arterial hypertension.

In the following days, we received the results of blood cultures for the first samples collected in the Hospital of Infectious Diseases, which confirmed an identification of *Capnocytophaga canimorsus*. The bacteriological culture of the sample collected in the Emergency Department was grown within a standard time of incubation and confirmed *Staphylococcus hominis* only, sensitive to standard antibacterials.

## Discussion and conclusions

The extreme invasiveness of *Capnocytophaga*, including *C. canimorsus*, and its propensity to cause septic shock has not yet been fully elucidated. However, we know several factors responsible for its virulence. It is known that *C. canimorsus* has a unique ability to induce saccharide lysis from the glycoproteins of human cells, including the phagocytes [11,12]. It can also break up long-chain molecules of glycogen and extract energy from the host’s energy supplies [12]. One of the polysaccharidases found on the surface of *C. canimorsus* can break N-Acetylglucosamine contained, among others, in human IgG [13]. Furthermore, it has been observed that *Capnocytophaga canimorsus* inhibits the bactericidal properties of macrophages, the phagocytosis of polymorphonuclear leukocytes and has a polysacharide structure on its surface which inhibits the activity of the complement system [[14], [15], [16]].

The presented case should prompt medical staff to remain vigilant in the management of dog or cat bite cases. Despite being rare, severe infection with *Capnocytophaga canimorsus* can be characterized by an exceptionally dramatic course of illness along with a high mortality rate [6,8,10]. Since *Capnocytophaga* culture grows slowly, in every case suspected of this infection, the laboratory should be informed about the need for extended incubation of the collected samples. In order to grow, *C. canimorsus* requires enriched agar media and blood cultures may be positive even after 14 days of incubation (6 days on average) [8].

In high-risk patients, i.e. those with anatomic or functional asplenia, hepatic cirrhosis, a history of alcohol abuse, taking immunosuppressive medications or with any other known immunity deficits, preventive antibacterial therapy should be considered if they are bitten by a dog or cat. Due to the pathogen’s sensitivity to beta-lactams, it seems reasonable to use amoxicillin with clavulanic acid as a first line treatment. In terms of treatment, including the treatment of severe infection, cephalosporins, carbapenems or finally piperacillin with tazobactam can be applied, as in the presented case. The onset of the treatment may be of crucial importance and whether or not the patient suffering from septic shock, with all its complications, will be cured is, of course, uncertain.

## Funding

This research did not receive any specific grant from funding agencies in the public, commercial, or not-for-profit sectors.

## CRediT authorship contribution statement

**Piotr Woźniak:** Conceptualization, Writing - original draft, Supervision. **Robert Szymczak:** Investigation, Writing - review & editing. **Agata Piotrowska:** Investigation, Writing - review & editing, Resources.

## Declaration of Competing Interest

None.
